# The Treatment of Burns

**Published:** 1901-10-12

**Authors:** 


### THE TREATMENT OF BURNS.

Many causes have conspired to render the treatment of burns unsatisfactory and disheartening to the surgeon who aims at a high standard of asepticism in his work. Owing to the intensity of the shock which is caused by injuries of this nature, by which the vital resistance of the patient is lowered, to the large area of skin involved—skin which under ordinary circumstances is generally more or less charged with pus-producing organisms—and to the filthy dressings with which anxious friends so often cover the injury before the arrival of the doctor, these wounds nearly always become septic, and in too many cases it happens that extensive burns remain an exception to the generally aseptic course of surgical cases. It becomes, then, a very important matter to see whether we cannot bring such burns and scalds within the fold of aseptic surgery.

Dr. Frederick Griffith,[1] of New York, in a very complete article on the subject of burns, enters a protest against the manner in which these injuries are too often treated, and after discussing the various methods which have been adopted from time to time in the management of such cases, urges the following as a reasonable manner of avoiding some of their evil consequences and of insuring, as far as possible, some degree of cleanliness in the wounds produced. First as to household remedies. Of these it is sufficient to say that they should not be used. Friends would do best by the patient, and would greatly assist the surgeon, if they would confine their efforts to carefully cutting away whatever clothing was loose about the burn and wrapping about it a piece of oiled or wax paper such as florists use, tied on with strips of muslin. A burn so handled is in the best condition for right surgical treatment. On removing the oiled paper protective the surgeon should with scissors and forceps cut away all shreds of burned clothing remaining about the wound, then he should puncture the blisters and drain away the fluid, for otherwise this will either be rapidly absorbed and set up febrile reaction, or it will coagulate and form a culture medium for pus-producing organisms. Infection being ever present in the skin, by trimming away all detached fragments a source of danger is avoided; therefore as much burned subcutaneous tissue as possible should also be cut away. A curette may be necessary, and when gently used will cause but little pain; but an anæsthetic may be required when large and deep burns are being dressed. Of course all the burned tissue cannot be removed at one dressing, and inflammation must assist the process, but we now know that in the processes by which dead and burned tissues are separated from the living the aid of pyogenic micro-organisms is not needed. In selecting an application for a burn, two principles are involved, the agent must cause no irritation and must be antiseptic, and Dr. Griffith strongly recommends the use of hydrogen dioxide. Having cleared up the wound then it is next to be thoroughly washed by syringing with a solution of hydrogen dioxide, one part to six of water. Much dead tissue will thus be dislodged and washed away, in addition to that removed with instruments. When foaming has practically ceased the wound must be dressed by applying all over its surface, and well over the edges to sound skin, strips of rubber tissue in sizes about ½ to ¾ of an inch wide, by 3 or 4 inches long, each strip to overlap the previous one laid down by a small margin. A few layers of loose sterile gauze are to be fluffed over the tissue, and the whole held in place by a gauze bandage, or by means of two or three narrow adhesive strips, and to secure perfect rest we must place the part in the best position to obtain muscular relaxation and support. Well padded splints may be used for the same purpose. There can be little doubt that burns treated as described by Dr. Griffith are likely to do better than when they are wrapped up in cotton wool, lint, and other fluffy material which sticks to the wound. For burns of moderate degree, however, picric acid seems so satisfactory an application that we may doubt whether it can be greatly improved upon. But when once discharge has been set up, we have no doubt about the utility of hydrogen dioxide, and for dressing nothing can be better than strips of protective freshly sterilised and applied much as described above, so as to allow free escape for all exuding fluid.

[1] New York Medical News, Aug. 24.

